# Towards Defining Nutrient Conditions Encountered by the Rice Blast Fungus during Host Infection

**DOI:** 10.1371/journal.pone.0047392

**Published:** 2012-10-10

**Authors:** Richard A. Wilson, Jessie Fernandez, Cristian F. Quispe, Julien Gradnigo, Anya Seng, Etsuko Moriyama, Janet D. Wright

**Affiliations:** 1 Department of Plant Pathology, University of Nebraska-Lincoln, Lincoln, Nebraska, United States of America; 2 School of Biological Sciences, University of Nebraska-Lincoln, Lincoln, Nebraska, United States of America; 3 Center for Plant Science Innovation, University of Nebraska-Lincoln, Lincoln, Nebraska, United States of America; Ghent University, Belgium

## Abstract

Fungal diseases cause enormous crop losses, but defining the nutrient conditions encountered by the pathogen remains elusive. Here, we generated a mutant strain of the devastating rice pathogen *Magnaporthe oryzae* impaired for *de novo* methionine biosynthesis. The resulting methionine-requiring strain grew strongly on synthetic minimal media supplemented with methionine, aspartate or complex mixtures of partially digested proteins, but could not establish disease in rice leaves. Live-cell-imaging showed the mutant could produce normal appressoria and enter host cells but failed to develop, indicating the availability or accessibility of aspartate and methionine is limited in the plant. This is the first report to demonstrate the utility of combining biochemical genetics, plate growth tests and live-cell-imaging to indicate what nutrients might not be readily available to the fungal pathogen in rice host cells.

## Introduction

Biotrophic fungi spend at least part of their lifecycle in the host cell without causing symptoms of disease and represent important intracellular pathogens of humans, animals, and plants. In particular, such fungi cause devastating diseases of crops [1], but long standing questions concerning which metabolites the fungi make themselves, and what they obtain from the plant, are largely unanswered [2]–[4]. Determining the metabolites available to pathogens in host tissue could reveal new information regarding pathogen-host interactions that would point the way to novel mitigation strategies.

The hemi-biotrophic ascomycete *Magnaporthe oryzae* is a serious threat to rice production and global food security [5]. Initial infection involves penetration of the host leaf by a specialized infection structure called the appressorium. The appressorium develops on the surface of the leaf and generates enormous internal turgor pressure that is directed onto a penetration hypha emerging from the base of the appressorium, forcing it through the surface of the leaf. The penetration hypha then forms a thin filamentous primary hypha that grows in the cell lumen before differentiating into bulbous invasive hyphae (IH). Successive biotrophic colonization of adjacent plant cells by IH proceeds for 4–5 days in susceptible cultivars before the fungus enters its necrotic phase [6], [7]. 10–30 % of global rice harvests are lost in this manner each year. How *M. oryzae* sustains growth during biotrophy, what constitutes the nutrient environment encountered during infection, and how accessible metabolites contribute to disease, is not known.

*M. oryzae* has extensive metabolic capabilities, growing axenically in synthetic 1% glucose minimal media (GMM) containing simple sources of nitrogen (*5*) and synthesizing all amino acids, purines and pyrimidines *de novo.* Moreover, *M. oryzae* carries genetic regulatory systems that allow it to respond dynamically to nutrient quality and quantity in the environment. These include nitrogen metabolite repression (NMR) and carbon catabolite repression (CCR), which ensure the utilization of preferred sources of nitrogen (ammonium and L-glutamine) and carbon (glucose), respectively [8], [9], [10]; and a Tor signaling pathway that might operate to regulate growth in response to nutrient availability [11]. Carbon and nitrogen metabolism is integrated in *M. oryzae* by the sugar sensor trehalose-6-phosphate synthase 1 (Tps1) [8]–[10], [12]. In response to glucose-6-phosphate (G6P) sensing, Tps1 stimulates NADPH production by increasing glucose-6-phosphate dehydrogenase (G6PDH) activity [8]. Elevated NADPH production inactivates a family of transcription factor inhibitor proteins, Nmr1-3 [9], resulting in CCR and the alleviation of NMR [10]. Tps1-dependent CCR ensures genes for utilizing alternative sources of carbon, such as cell wall polysaccharides, are not expressed in the early, biotrophic stage of infection when G6P is likely abundant in host tissue. In addition, Tps1 control of NMR ensures genes for metabolizing alternative sources of nitrogen can be expressed under the nitrogen limiting conditions that might be found in the nutrient poor apoplast [3] if G6P is present, but are not expressed if G6P is absent [10]. This is important because some *M. oryzae* virulence-associated genes are expressed in axenic cultures under conditions of nitrogen starvation [13], [14], and at least two of these – *SPM1* encoding a serine protease [14] and *PTH11* encoding a plasma membrane protein [15] – are under Tps1control [10]. Thus, Tps1 control of NMR and CCR could provide a mechanistic framework for understanding how virulence genes are expressed early in infection (when the fungus might be in a glucose-rich, nitrogen-poor environment such as might be found in the host apoplast), and how genes for utilizing alternative carbon sources are derepressed later in infection (when the fungus might be in a glucose-poor environment as colonized cells expire and necrotrophy commences). However, a major impediment to validating this model is a poor understanding of the actual nutrient conditions encountered by *M. oryzae* during infection, what nutrients can be acquired from the host, and how closely axenic growth in synthetic minimal media mimics the nutrient conditions of the plant.

We seek to address this deficit in our knowledge and here reason that generating auxotrophic mutants of *M. oryzae*, and observing how they grow on supplemented plate tests compared to *in planta* colonization, would afford us new insights into the identity of available nutrients during infection and inform us of the metabolic status of both host and pathogen. As proof-of-principle, we report the construction and characterization of a methionine auxotrophic mutant of *M. oryzae* that can form functional appressoria but cannot establish disease. By comparing remediation of *ex planta* axenic growth with live-cell-imaging of *in planta* colonization, we show that *de novo* methionine biosynthesis is essential for the cell-to-cell movement of IH. Consequently, we have identified for the first time some of the nutrients not readily accessible to the pathogen during infection.

## Results

### *MoSTR3* gene replacement mutants are unable to convert homocysteine to methionine

**Figure 1:**
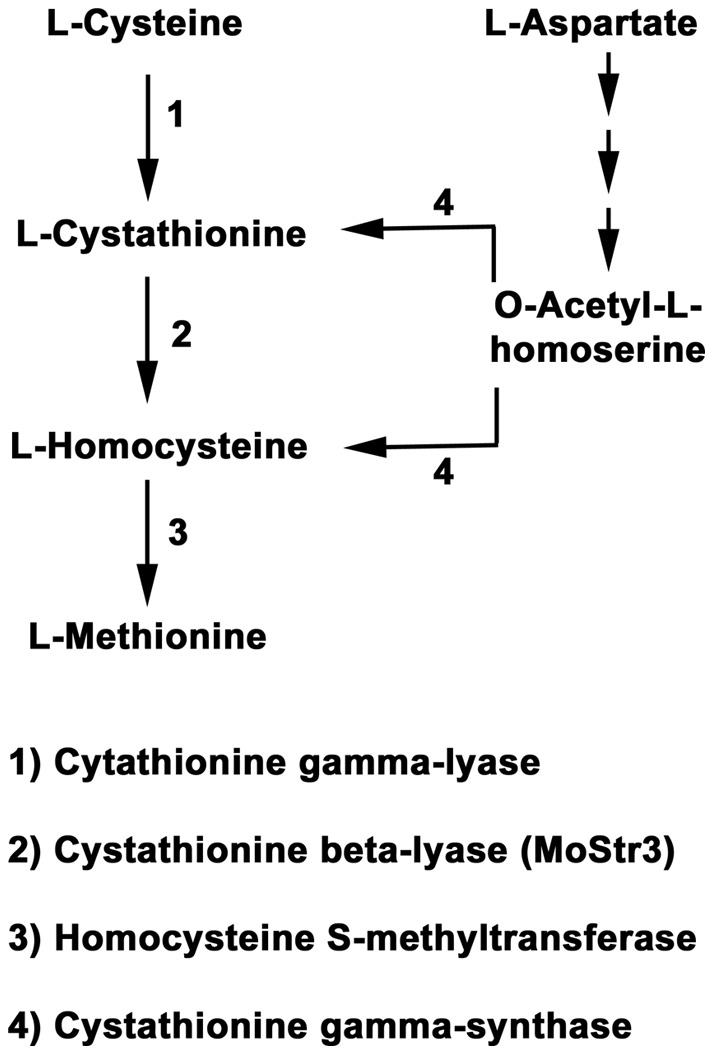
Methionine metabolism in *Magnaporthe* oryzae. *De novo* biosynthesis of methionine requires homocysteine derived from cysteine – via cystathionine – and involves cystathionine beta-lyase (MoStr3). Homocysteine might also result from O-acetyl-L-homoserine. O-acetyl-L-homoserine is derived from aspartate in a pathway involving a number of enzymatic steps that have been omitted for clarity [16]. This scheme is based on the predicted methionine and cysteine metabolic pathway map for *M. oryzae* at the Kyoto Encyclopedia of Genes and Genomes.

**Figure 2:**
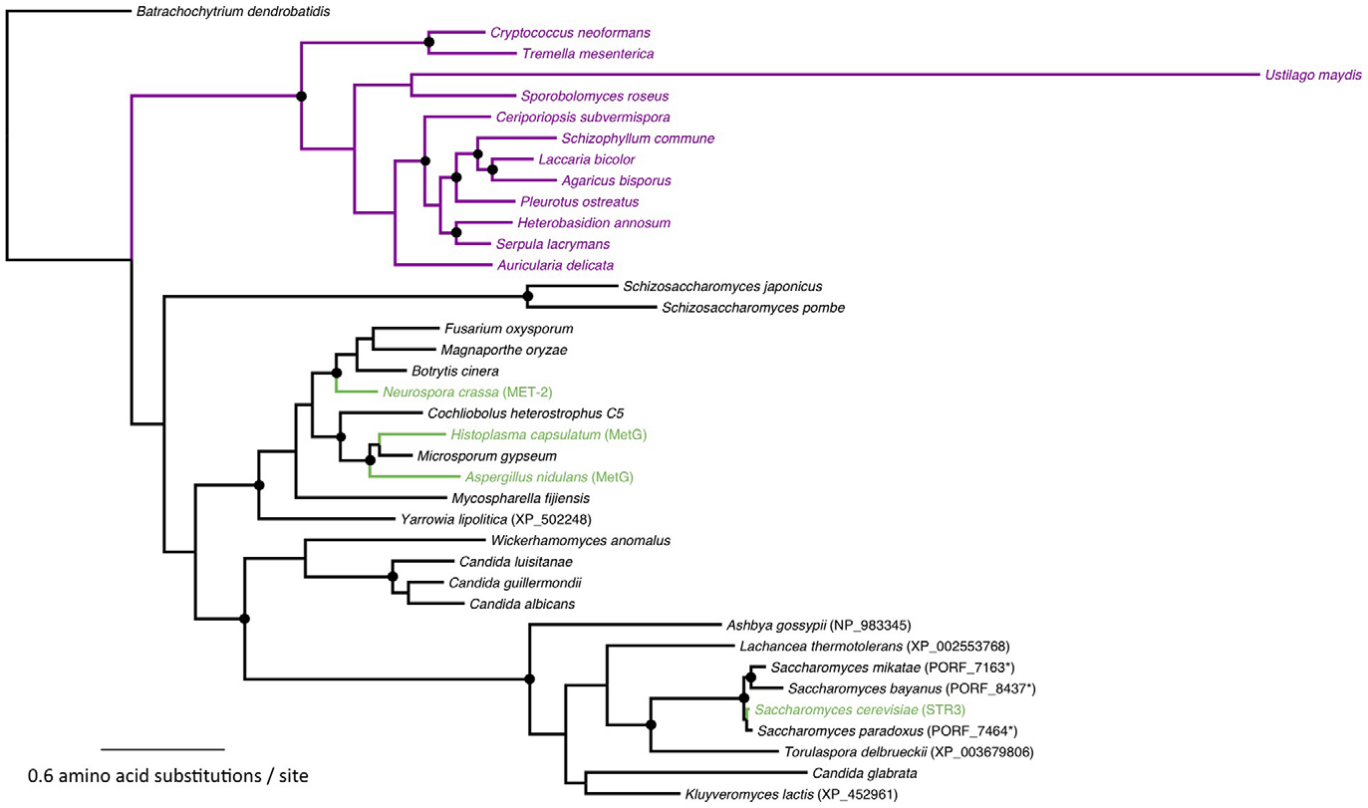
Maximum likelihood phylogeny of STR3 orthologs. The maximum likelihood phylogeny was reconstructed with RAxML, as described in Materials and Methods. Nodes with black circles indicate that these clusters are well supported (≥70% bootstrap support). Purple branches and species names indicate sequences with a fused C-terminal mevalonate kinase domain. Species that have known STR3 orthologs prior to this study are shown in green. Protein sequences were obtained from the Fungal Genome Collection (FGC). For those species not present within FGC, sequences were obtained from the NCBI database and their accession numbers are given in parentheses. Asterisks indicate sequences retrieved from the Saccharomyces Genome Database (SGD).

The predicted pathway for methionine biosynthesis in *M. oryzae* is shown in Figure 1. We chose to analyse this pathway in *M.* oryzae because the genes and enzymes involved have been extensively studied using classical and molecular genetics in the yeast *Saccharomyces cerevisiae* and the filamentous fungi *Aspergillus nidulans* and *Neurospora crassa* ([16]–[18]; and references therein). *metG* in *A. nidulans* [17], *met-2* in *N. crassa* [19] and *STR3* in *Saccharomyces cerevisiae* [20] are orthologous genes encoding cystathionine beta-lyase (EC:4.4.1.8) that converts cystathionine to homocysteine during the *de novo* biosynthesis of methionine. When these genes are deleted, the resulting mutant strains are strict methionine auxotrophs. Wild type growth and development is restored in strains lacking a functional cystathionine beta-lyase when grown on media supplemented with methionine, indicating STR3 proteins have no additional roles in the cell unrelated to methionine biosynthesis. We used this information to determine which gene to target in order to abolish methionine metabolism. Figure 2 shows that STR3 orthologues are widely distributed across fungal taxa. While the Ascomycota carry similar STR3 orthologues, as shown in Figure 2, STR3 orthologues identified in all the Basidiomycota we examined (except for *Postia placenta*) carry extra C-terminal sequences (∼450 aa) compared to the ascomycete STR3 orthologues. This extra region has weak sequence similarity (30–40% identity) with mevalonate kinase. Mevelonate kinase proteins are found in a wide variety of eukaryotes and prokaryotes, but among fungi they exist only in the Ascomycota (e.g., XP_723495.1 from *Candida albicans*, XP_661473.1 from *A. nidulans* and ERG12 from *S. cerevisiae* [21]) where, at least in *S. cerevisiae*, they function in the biosynthesis of isoprenoids and sterols [21]. Interestingly, our preliminary analysis indicates that this mevalonate kinase sequence does not exist in basidiomycetes as a stand-alone protein nor as a domain as part of other proteins (see Materials and Methods). The significance of this sequence to STR3 enzyme function, sterol biosynthesis and/or basidiomycete lifestyle remains to be functionally determined, but we demonstrate here how comparative genomics can yield new insights into well-studied and classically determined metabolic pathways, warranting further comparisons of other biochemical enzymes across a wide range of fungal taxa.

**Figure 3:**
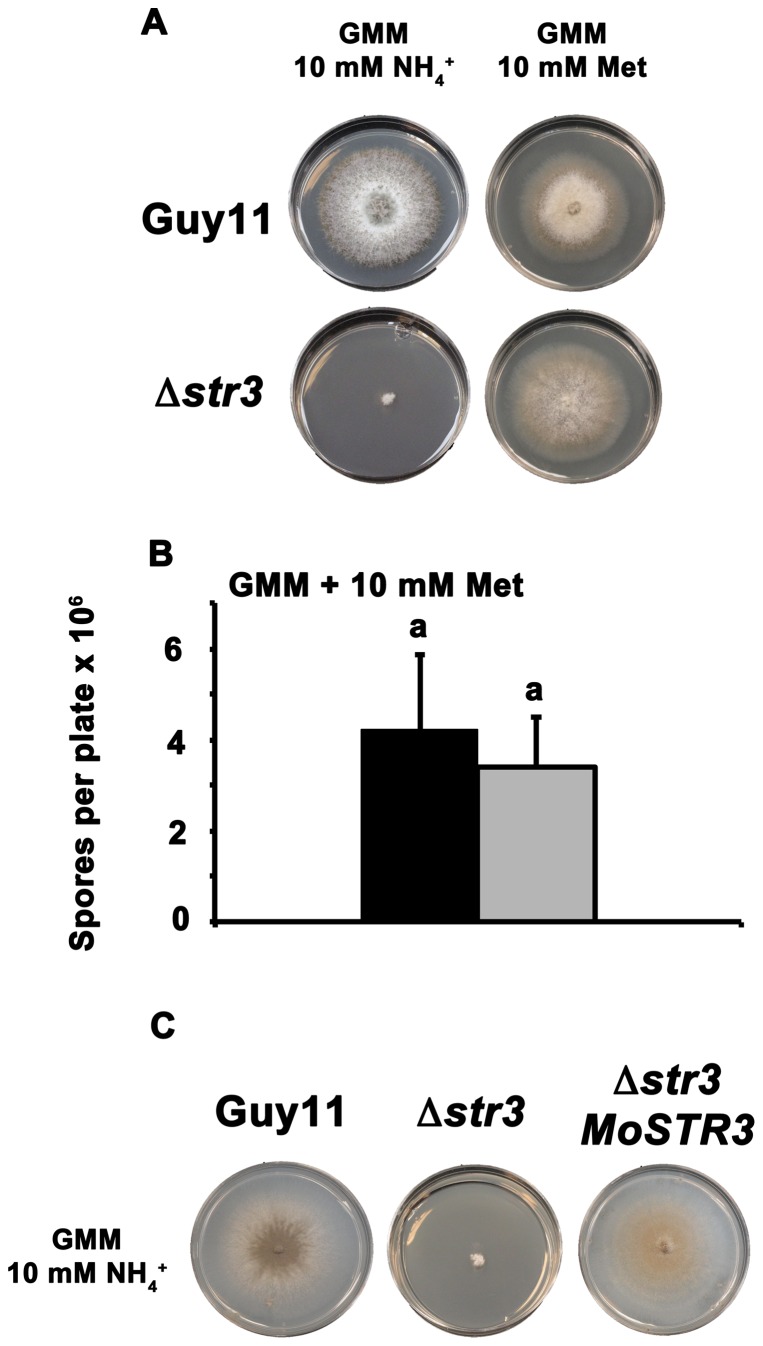
Deleting *MoSTR3* from the *M. oryzae* genome results in a strict requirement for exogenous methionine. (**A**) Growth of Δ*str3* strains was tested on 1% glucose minimal media (GMM) with the indicated sole nitrogen sources added to a final concentration of 10 mM. As predicted from Figure 1, Δ*str3* strains cannot grow on minimal media unless methionine is used as the sole nitrogen source. (**B**) If methionine is supplied in the media (as sole nitrogen source at 10 mM concentration), Δ*str3* strains (grey bar) sporulate like wild type Guy11 (closed bar). Spore counts were determined following 14 days of growth using 5 independent replicates for each strain. Bars with the same letters are not significantly different (*Student's t-test* p≤0.05). Error bars are standard deviation. (**C**) Transforming methionine-requiring Δ*str3* strains with a wild type copy of *MoSTR3* complements the Δ*str3* genetic lesion and allows growth of Δ*str3 MoSTR3* strains on minimal media lacking methionine. All plate images were taken after 10 days growth.

Figure 2 shows *M. oryzae* carries one copy of a putative cystathionine beta-lyase encoding gene, which we have named *MoSTR3* (MGG_07074, [22]) (Figure 1). *MoSTR3* was chosen as a likely candidate for a gene encoding a methionine biosynthetic enzyme with no roles in additional cellular processes. We used high-throughput, established PCR-based protocols to replace the coding region of *MoSTR3* with the hygromycin B resistance selectable marker, *hph* [9]. The resulting Δ*str3* deletion strains were abolished for growth on GMM media with ammonium (NH_4_^+^) as the sole nitrogen source but grew like wild type Guy11 strains on GMM with methionine as the sole nitrogen source (Figure 3A). Δ*str3* strains also sporulated like wild type strains on GMM containing methionine (Figure 3B). Thus, although abolished for growth on GMM lacking methionine, spore production of Δ*str3* strains was not significantly different to Guy11 on GMM containing methionine as the sole nitrogen source (*Student's t-test* p = 0.42). This indicates that the role of *MoSTR3* in growth and development appears to lie solely in its methionine biosynthetic function. Moreover, the uptake and utilization of exogenous methionine is not impaired in Δ*str3* strains compared to Guy11.

To ensure the phenotype of Δ*str3* strains arises solely from deletion of *MoSTR3*, a full-length copy of *MoSTR3* including promoter and terminator sequences was reintroduced into Δ*str3* strains. Figure 3C shows the resulting Δ*str3 MoSTR3* complementation strains were restored for growth on minimal media lacking methionine (Figure 3C).

**Figure 4:**
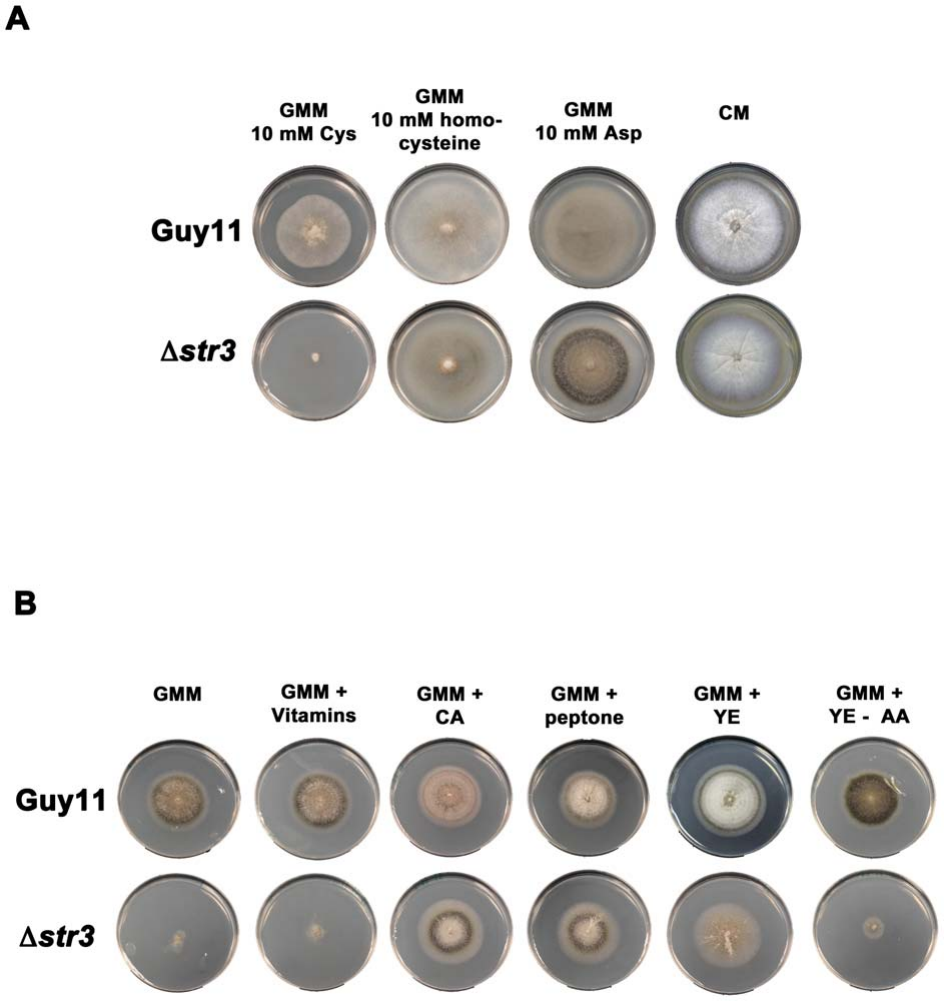
Growth characteristics of a methionine-requiring mutant strain of *M. oryzae*. (**A**) In addition to media containing methionine, Δ*str3* strains can also grow on media containing homocysteine but not cysteine as sole nitrogen source (10 mM). Plate tests also suggest a cystathionine beta-lyase-independent pathway operates in *M. oryzae* to produce homocysteine from O-acetyl-L-homoserine, resulting in the growth of Δ*str3* strains on plates containing aspartate. Growth of Δ*str3* strains on complete media (CM) requires no additional supplementation. (**B**) Supplements found in CM, such as vitamins, casamino acids (CA), peptone, yeast extract (YE) and yeast nitrogen base without amino acids (YE-AA), were added in the same amounts to GMM (containing 10 mM NH_4_^+^) to determine what component(s) of CM remediated Δ*str3* growth.

Confirming that *MoSTR3* encoded a likely cystathionine beta-lyase, we found Δ*str3* strains could grow like Guy11 on GMM with homocysteine as a sole nitrogen source but were unable to grow on cysteine (Figure 4A), indicating a defect in the conversion of cysteine to homocysteine. A previous report concluded homocysteine was toxic to *M. oryzae* when metabolized as a sole sulphur source [23]. We did not observe the same level of toxicity when homocysteine was used as a sole nitrogen source, likely because GMM contains an alternative sulphur source (magnesium sulphate).

We next tested the growth of Δ*str3* strains on a wide range of amino acids (not shown) and found in addition to methionine and homocysteine, aspartate could also remediate the growth of Δ*str3* strains on GMM in the absence of methionine (Figure 4A). Homocysteine can be synthesized from aspartate via O-acetyl-L-homoserine in a cystathionine beta-lyase-independent pathway [16], and this pathway might be activated in *M. oryzae* in the presence of exogenous aspartate (Figure 1). Thus, aspartate can be a suppressing nitrogen source for methionine – requiring Δ*str3* strains.

We also observed Δ*str3* strains were capable of growth on undefined complete media (CM, Figure 4A). Compared to GMM, CM contains complex supplements such as vitamins and peptides derived from partial protein digestion. To determine which component(s) of CM remediated growth of Δ*str3* strains, we added each supplement found in CM separately to GMM and discovered that sources of peptides and amino acids, but not vitamins or yeast extract without amino acids, allowed growth of Δ*str3* strains (Figure 4B).

Taken together, these results suggest Δ*str3* strains are impaired in the conversion of cysteine to homocysteine, via cystathionine, during *de novo* methionine biosynthesis. Growth media containing methionine, homocysteine, aspartate, or complex mixtures of peptides that likely supply these amino acids, result in wild type growth and development of Δ*str3* mutant strains.

### Δ*str3* mutant strains are severely reduced in pathogenicity compared to wild type

**Figure 5:**
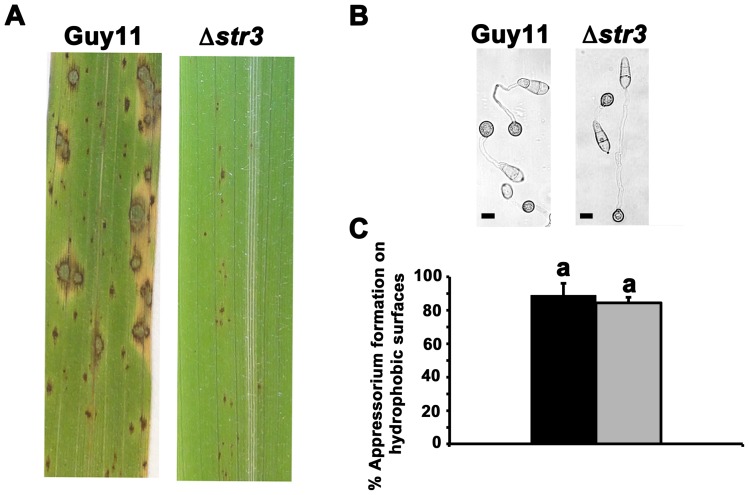
Disrupting *MoSTR3* function abolishes infection but not appressorium formation. (**A**) Δ*str3* strains cannot infect rice leaves. Conidial spores were applied to three-week old plants of the susceptible cultivar CO-39 at a rate of 5×10^4^ spores ml^−1^. Images were taken after 144 hpi. (**B**) Although unable to infect rice leaves, Δ*str3* strains could form appressoria on hydrophobic plastic cover slips, shown here after 24 hr. Scale bar is 10 µm. (**C**) The rate of appressorium formation for Δ*str3* strains was compared to Guy11. Three plastic coverslips per strain were inoculated with 200 µl of a spore suspension containing 1×10^5^ spores ml^−1^. After 24 hr, the number of appressoria formed by 50 conidia was counted on each cover slip, and a mean value generated. Error bars are standard deviation. Closed bars are values for Guy11 and grey bars are values for Δ*str3* strains. Bars with the same letter are not significantly different (*Student′s t-test* p≤0.05).

To understand the effects of the Δ*str3* mutation on infection, we inoculated intact rice leaves with spores of the wild type Guy11 and Δ*str3* strains and found that the methionine-requiring mutant strains were severely attenuated in their ability to form spreading necrotic lesions at 144 hours post inoculation (hpi; Figure 5A). Δ*str3 MoSTR3* complementation strains were restored in their ability to infect rice (Figure S1), indicating the infection defect shown in Figure 5A results solely from the loss of *MoSTR3* function.

### Δ*str3* strains are not impaired in appressorium formation

To determine if loss of pathogenicity was due to impaired appressorium development in Δ*str3* strains, we inoculated conidial spores of Guy11 and Δ*str3* onto hydrophobic plastic coverslips that are used to induce appressorium formation in the laboratory [7]. After 24 hr, Figure 5B shows that both strains had formed normal-looking appressoria on the inductive surfaces, suggesting Δ*str3* strains are not delayed or impaired in appressorium development compared to the Guy11 wild type strain. We also determined the rate of appressorium formation for each strain by counting how many appressoria had formed from 50 conidia after 24 hr on the inductive surfaces. This was repeated in triplicate for each strain. Figure 5C shows that the percentage of conidia that had formed appressoria after 24 hr on hydrophobic inductive surfaces was not significantly different (*Student′s t-test* p  = 0.42) between Guy11 and Δ*str3* strains. Thus, *de novo* methionine biosynthesis is not required for appressorium formation or development but is essential for pathogenicity.

### Δ*str3* strains can penetrate rice leaf cuticles but IH growth is restricted in host cells

**Figure 6:**
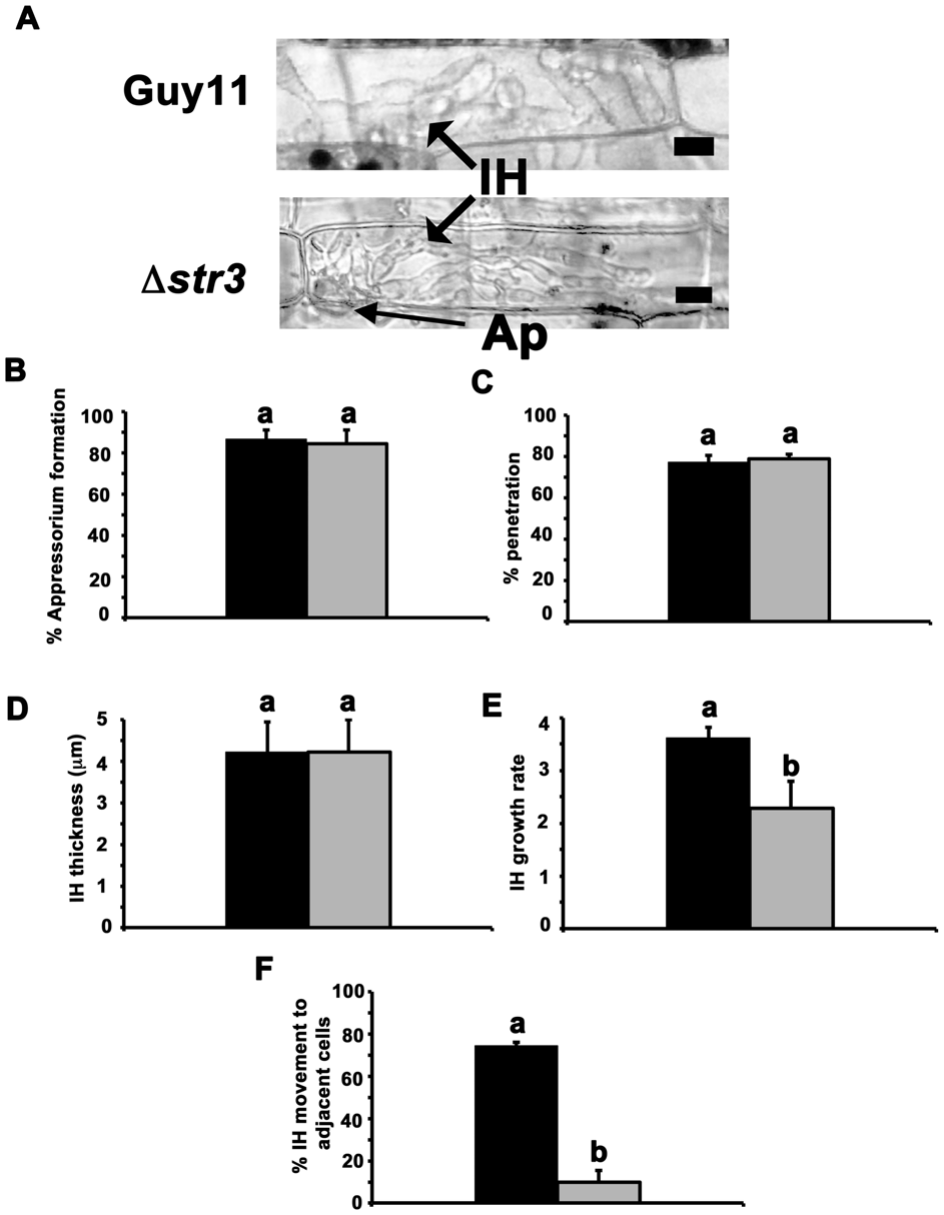
Invasive hyphal growth but not appressorium formation, penetration or IH elaboration is restricted in Δ*str3* strains. (**A**) Live-cell-imaging of rice leaf sheaths, 48 hpi, show that both Guy11 and Δ*str3* strains can elaborate IH in infected cells. Ap is appressorium on the surface of the leaf and its position is indicated to demonstrate that IH growth occurs in the plant cell and not epiphytically on the surface of the leaf. Scale bar is 10 µm. (**B**) The mean rate of appressorium formation on the rice leaf surface was determined using the rice leaf sheath assay. 5×10^4^ spores ml^−1^ of each strain were inoculated onto rice cuticles as described in Materials and Methods. At 36 hpi, images were taken, and the number of appressoria that had developed from 50 conidia of each strain was analyzed. This was repeated three times for each strain to determine the mean value. Error bars are standard deviation. Closed bars are values for Guy11 and grey bars are values for Δ*str3* strains. Bars with the same letter are not significantly different (*Student's t-test* p≤0.05). (**C**) Rate of appressorium penetration. The number of appressoria that had penetrated the leaf cuticle and developed visible IH, from a total of 50 appressoria observed, was determined at 36 hpi. The average was determined from three independent replicates. Error bars are standard deviation. Closed bars are values for Guy11 and grey bars are values for Δ*str3* strains. Bars with the same letter are not significantly different (*Student′s t-test* p≤0.05). (**D**) The thickness of IH for each strain was measured. Three leaf sheaths were inoculated with each strain, and 50 IH widths, arising from 50 individual appressoria on the surface, were determined for each replicate at 48 hpi to generate a mean IH width value. Error bars are standard deviation. Closed bars are values for Guy11 and grey bars are values for Δ*str3* strains. Bars with the same letter are not significantly different (*Student's t-test* p≤0.05). (**E**) IH growth was determined for each strain, at 48 hpi, using the four-point scale developed by Saitoh et al [24]. According to this scale, 1 =  IH length shorter than 10 µm with no branching; 2 =  IH length is 10–20 µm with 0–2 branches; 3 =  IH length is longer than 20 µm and/or with more than 2 branches within one cell; 4 =  IH has spread to adjacent cells. Three leaf sheaths were inoculated with each strain, and 50 IH growth rates were determined for each replicate to generate a value for the mean IH growth rate. Error bars are standard deviation. Closed bars are values for Guy11 and grey bars are values for Δ*str3* strains. Bars with the same letter are not significantly different (*Student′s t-test* p≤0.05). (**F**) IH movement to adjacent cells is curtailed in Δ*str3* strains. Three leaf sheaths were inoculated with each strain, and the movement of IH into adjacent cells at 48 hpi was observed from a total of 50 primary infected cells per replicate to generate a value for the mean proportion of IH that had moved to adjacent cells (growth level 4). Error bars are standard deviation. Closed bars are values for Guy11 and grey bars are values for Δ*str3* strains. Bars with the same letter are not significantly different (*Student's t-test* p≤0.05).

Although not required for appressorium formation, we next considered whether *de novo* methionine biosynthesis might be important for appressorium function. Applying live-cell-imaging techniques [6] to epidermal rice cells, we discovered both Guy11 and Δ*str3* strains were able to gain entry to host cells (Figure 6A), suggesting appressorium function is not compromised in Δ*str3* strains. This prompted us to use the rice leaf sheath assay to quantify how the infection process compared and contrasted between Guy11 and Δ*str3* strains in order to understand why methionine-requiring mutants were attenuated in pathogenicity (Figure 6B–6F). First, we confirmed that the rate of appressorium formation was not significantly different (*Student's t-test* p = 0.44) between Guy11 and Δ*str3* strains on rice surfaces (Figure 6B, measured after 36 hpi). Next, we determined that, at 36 hpi, most of the appressoria in Guy11 and Δ*str3* strains that formed on the rice surface had been successful in penetrating the leaf cuticle (Figure 6C), confirming that appressoria function is not significantly affected (*Student′s t-test* p = 0.44) in Δ*str3* strains compared to Guy11. Thus, *de novo* methionine biosynthesis is not required for appressorium function.

Figure 6D shows that the average width of IH at 48 hpi (measured from 50 individual hypha, in triplicate) was also not significantly different (*Student's t-test* p = 0.95) between Guy11 and Δ*str3* strains. Therefore, the elaboration of bulbous IH from primary hyphae in host cells following penetration does not require *de novo* methionine biosynthesis.

Taken together, Figure 5 and Figure 6A–D show that *de novo* methionine biosynthesis is not required for appressorium formation, penetration, or the elaboration of IH within host cells. However, differences between Guy11 and Δ*str3* strains began to emerge when we looked at the growth of IH in host cells. Recently, Saitoh and associates [24] characterized the IH growth of a *M. oryzae* mutant, lacking the secreted protein MC69 required for pathogenicity, using a four-point scale, with 1 the lowest and 4 the highest level of growth. We characterized IH growth for Guy11 and Δ*str3* strains using this scale, and generated a mean value for growth rate using 50 IH growth measurements for each strain, in triplicate. Figure 6E shows IH growth, at 48 hpi, is significantly reduced (*Student′s t-test* p  =  0.03) in Δ*str3* strains (mean growth rate  = 2.3±0.5) compared to Guy11 (mean growth rate  = 3.6±0.2). When we focused on the proportion of each strain that had achieved growth level 4, indicating IH have spread to adjacent cells, we found at 48 hpi that approximately 70% of Guy11 IH had moved from the primary infected cell (ie the cell first penetrated by the appressorium) to adjacent cells, but only 10% of Δ*str3* IH was found growing beyond the primary infected cell (Figure 6F). Thus the movement of IH into cells adjacent to the primary infected cell, at 48 hpi, was significantly constrained in Δ*str3* strains compared to Guy11 (*Student′s t-test* p = 0.0001). This inhibition of Δ*str3* IH growth – either in the primary infected cell (Figure 6E) or between adjacent cells (Figure 6F) – reflects the reduced lesion sizes shown in Figure 5A.

Comparing plate growth tests, development and *in planta* growth of Δ*str3* and Guy11 strains (Figures 3–6), we conclude *de novo* methionine biosynthesis is essential for IH growth. In addition, because exogenous sources of methionine and aspartate remediate Δ*str3* growth and development on plates, we suggest that during infection, *M. oryzae* does not have extensive access to free aspartate or methionine in the plant, nor does the biotrophic fungal stage encounter a rich milieu of plant-derived peptides that can liberate aspartate or methionine to remediate Δ*str3* growth. This suggests infection occurs in a nitrogen poor environment – at least from the perspective of the fungus, where nitrogen stores in the plant might initially be inaccessible to the invading pathogen.

## Discussion

The hemi-biotrophic rice blast fungus *M. oryzae* has global regulatory systems which allow genetic responses to available carbon and nitrogen sources in the host, but what those sources are is largely unknown. Genetic evidence [10] and genome-wide transcriptional studies [14], [25] suggest early infection might occur under nitrogen starvation conditions, but the content and abundance of nitrogenous compounds encountered during biotrophy is understudied [4]. The goal of this work was to understand what genetic approaches could be developed to determine the available or accessible nutrient content of host plants during infection. We reasoned that biochemical mutants, requiring nutrient supplementation for growth on plates, would only establish infection in plants if they received the same nutrient(s) from the host. Conversely, biochemical mutants that could not access the required nutrients in the host would enter the plant but fail to establish disease. As proof-of-principle, we generated a methionine auxotrophic mutant strain of *M. oryzae*, Δ*str3*, which had a strict requirement for methionine or aspartate on GMM (homocysteine supported somewhat poorer growth of both Guy11 and Δ*str3* strains on GMM). In addition, nitrogenous components of CM, including sources of partially digested peptides, also permitted growth. When applied to plants, Δ*str3* strains were greatly reduced in lesion development, although live-cell-imaging demonstrated they produced functional appressoria and could invade host cells. However, Δ*str3* strains were unable to sustain invasive hyphal growth in rice cells and largely failed to progress to cells adjacent to the point of infection. This suggests both that Δ*str3* strains are unable to obtain sources of methionine or aspartate during infection, and that the limited growth of Δ*str3* strains in host plant cells represents the point at which endogenous methionine and aspartate sources might be finally exhausted following the successful formation of appressorium, plant penetration and IH elaboration. These observations are consistent with the appressorial transcriptional profiling studies of Soanes and co-workers [26] that suggested amino acid uptake is not a significant process during appressorium development and the initial stages of plant infection, and also supports recent work which showed proteasome-mediated turnover of endogenous proteins is required for appressorium development and pathogenicity in *M. oryzae* [27].

Taken together, this work demonstrates that *de novo* methionine biosynthesis is essential for infection because the Δ*str3* suppressing metabolites shown in Figure 3 and 4 – such as free sources of aspartate and methionine or mixtures of peptides – are not readily available to the fungus during at least the first- and second-cell stage of biotrophic infection. This is the first account to indicate what metabolites the plant does not provide *M. oryzae* during colonization, thus shedding light on both plant host and fungal pathogen metabolism. This study also demonstrates the utility of combining biochemical genetics with live-cell-imaging to answer fundamental questions regarding the host cell nutrient environment.

## Materials and Methods

### Strain growth conditions and physiological tests

The strains used in this study were derived from Guy11 and maintained as filter stocks at −20°C in the Wilson laboratory. Strains were grown on complete medium (CM) containing 1% (W/V) glucose, 0.2% (W/V) peptone (Difco), 0.1% (W/V) yeast extract (Difco), 0.1% (W/V) casamino acids (Difco) and 0.001% (V/V) vitamin solution [containing 0.01% (W/V) each of biotin, pyridoxin, thiamine, riboflavin, PABA and nicotinic acid (Sigma)]; and on 1% glucose minimal medium (GMM) with 10 mM NH_4_^+^ as sole nitrogen source (unless otherwise stated) and containing 0.52 g/l KCl, 0.52 g/l MgSO_4_7H_2_O, 1.52 g/l KH_2_PO_4_, 0.001% (W/V) thiamine and 0.1% (W/V) trace elements (Fisher). CM supplements were added to GMM at the same concentrations as for CM. Amino acids and homocysteine (Sigma) were added to GMM as sole nitrogen sources at a final concentration of 10 mM. To inoculate plates, filter stocks were revived on CM and 10 mm^2^ blocks of mycelium were transferred to the center of each plate. Strains were grown for 10–16 days at 26°C with 12 hr light/dark cycles. After 10 days of growth, plate images were taken with a Sony Cyber-shot digital camera, 14.1 mega pixels. Fungal spores were counted on a haemocytometer (Corning) following harvesting in sterile distilled water from 14-day-old plates. For appressorial development assays, 200 µl of a 1×10^5^ spores ml^−1^ spore suspension was added to plastic coverslips mounted on a glass slide support and placed in a glass dish, with moisture, for 24 hr. Rates of appressorium formation were determined by counting the number of appressoria formed by 50 conidia after 24 hr. This was repeated three times for each strain.

### Analysis of the distribution of STR3 orthologues across the fungal kingdom

We used the Fungal Genome Collection (FGC) website (http://bioinfolab.unl.edu/emlab/FGC/) to search through the STR3 (MGG_07074) orthologues from 81 genomes across the fungal kingdom. The specific description and screen-shots detailing this analysis can be found in the “Use Cases” page of the FGC website. Further confirmation was done by reciprocal BLAST [28] between *S. cerevisiae* and other fungal genomes; potential orthologue(s) in each species was queried against the *S. cerevisiae* genome, returning STR3 as the highest scoring hit. We confirmed that each genome contained a single copy of the *STR3* orthologue.

Protein sequences of orthologue candidates were aligned using MAFFT [29]. Nearly all STR3 orthologues displayed high sequence similarity (>60%) and coverage (>85%) except for the extra C-terminal region (mevalonate kinase domain) found in the Basidiomycota as mentioned above. Phylogenetic analysis was done using RAxML version 7.0.4 [30] with the WAG substitution matrix and the gamma distribution parameter estimated. Bootstrap analysis for branch support was done with 1000 pseudoreplicates. The alignment and phylogenetic analysis were done both with and without the extra C-terminal region (the mevalonate kinase domain mentioned above).

The STR3 orthologues found in basidiomycetes included extra ∼450 amino acids in their C-terminal regions (see the “Use Cases” page of the FGC website). These sequences were examined by similarity search using BLASTP against the non-redundant (NR) database from the National Center for Biotechnology Information (NCBI: http://blast.ncbi.nlm.nih.gov/). These extra sequences were found to be similar to mevalonate kinase (*e.g.*, NP_013935 in *S. cerevisiae*, NP_000422.1 in human, and NP_198097.1 in *Arabidopsis thaliana*). Mevalonate kinase is found in both eukaryotes and prokaryotes, and the basidiomycete sequences are equally distant (30–40% identity) from mevalonate kinase proteins found in ascomycetes, metazoans, and plants. In basidiomycetes, the sequences similar to mevalonate kinase exist only as part of the cystathionine beta-lyase orthologues. We found no other mevalonate kinase homologues as stand-alone proteins. On the contrary, in ascomycetes, we found mevalonate kinases only as stand-alone (single-domain) proteins (*e.g.*, NP_013935 in *S. cerevisiae*). On each ascomycete genome, the genes encoding cystathionine beta-lyase and mevalonate kinase do not appear to be clustered together.

### Targeted gene replacement

Protoplast generation and transformation were performed as described previously [31]. DNA for PCR was extracted from Guy11 strains as described previously [8]. Gene replacement of *STR3* by the hygromycin phosphotransferase-encoding gene *hph* employed the PCR-based split marker method described in [9]. The *STR3-*specific primers used were as follows: Str3NesF: CATCGCTATTGCAAAAATAACCTGG and Str3-2: GTCGTGACTGGGAAAACCCTGGCG GCCCGCCATGATGACTCGTC to amplify 1 KB of the right flank of *STR3*; Str3-3: TCCTGTGTGAAATTGTTATCCGCT GATACCCCACGAATAGACGCAAAAG and StrNesR: TCGGGGAGCACACAACTGGC to amplify 1 KB of the left flank of *STR3*; Str3-1: AAGTATTTTTTGCCAACAGCCGG and Str3-4: GAACACATTAGCCACCGCACTTCC were used to screen for homologous recombination resulting in *MoSTR3* gene replacement. PCR conditions used annealing temperatures of 65°C with extension times of 2 minutes. M13 sequences used to fuse the flanks to *hph* are underlined. We studied three independent mutants resulting from gene replacement of *MoSTR3* and all had the same phenotype with regards to being unable to grow on GMM lacking methionine or being unable to establish disease. To determine that deletion of *MoSTR3* resulted in the observed phenotype, we complemented one of our Met-deficient mutant strains with the full length coding sequence of *MoSTR3*. *MoSTR3* was amplified from wild type DNA with the primers metG190bpF (TCACTCAATTACAGTACAAAGTCGCAAGA) and metG341bpR (ACCAGTGGAACACGTTTGACCTTTT), using the PCR conditions described above, to generate a PCR product containing the *MoSTR3* coding sequence flanked by 190 bp of upstream sequence and 341 bp of downstream sequence. The PCR product was cloned into pGEM-T Easy (Promega, USA) and either the *MoSTR3* coding region in pGEM-T, or the *MoSTR3* PCR product alone, was transformed into protoplasts of Δ*str3*. Transformants restored for methionine prototrophy were selected on GMM with 10 mM NH_4_^+^ as sole nitrogen. 10 to 15 transformants, restored for methionine prototrophy, were obtained per selection plate when Δ*str3* strains were transformed with the full length *MoSTR3* coding sequence, but no methionine prototrophs were obtained on the same media when Δ*str3* strains were transformed with an empty pGEM-T vector. Δ*str3 MoSTR3* complementation strains remained hygromycin resistant, indicating random insertion of the full-length STR3 coding sequence had occurred in the Δ*str3* genome, and were confirmed by PCR. All Δ*str3 MoSTR3* complementation strains were re-screened for methionine prototrophy, and two complementation strains resulting from transformation of Δ*str3* with the *MoSTR3* PCR product were applied to plants to show they were restored for pathogenicity (one of which is shown in Figure S1).

### Rice plant infections and live-cell-imaging

Rice plant infections were made using a susceptible dwarf Indica rice (*Oryza sativa*) cultivar, CO-39, as described previously [9]. Fungal spores were isolated from 12–14 day-old plate cultures and spray-inoculated onto rice plants of cultivar CO-39 in 0.2% gelatin at a concentration of 5×10^4^ spores ml^−1^, and disease symptoms were allowed to develop under conditions of high relative humidity for 96–144 hrs. Live-cell-imaging was performed as described in [6] also using the susceptible rice cultivar CO-39. Briefly, 3 cm-long sheath segments from 3–4 week-old rice plants were placed in a glass container with a wet paper towel for high humidity conditions. Sheaths were kept horizontal and flat in a stable support to avoid contact with the wet paper. By using a pipette, a spore suspension of 5×10^4^ spores ml^−1^ in 0.2% gelatin was injected in one end of the sheath. The suspension was uniformly distributed inside the sheaths. After 36 and 48 hpi, the sheath ends were removed and the segments were trimmed and immediately observed under the microscope. Images were taken using a Nikon Eclipse 50i microscope and a Nikon D100 digital net camera.

## Supporting Information

Figure S1 **Δ** ***str3*** **strains complemented with** ***MoSTR3*** **are restored for pathogenicity.** Spores of wild type Guy11 strains and Δ*str3 MoSTR3* complementation strains were applied to three-week old rice plants of the susceptible cultivar CO-39 at a rate of 5×10^4^ spores ml^−1^. Images were taken after 144 hpi.
(TIF)
