# Supplementary figures and images for: Towards Defining Nutrient Conditions Encountered by the Rice Blast Fungus during Host Infection
Source: PLoS One. 2012 Oct 10;7(10):e47392. doi: 10.1371/journal.pone.0047392 (PMC3468542; doi:10.1371/journal.pone.0047392)

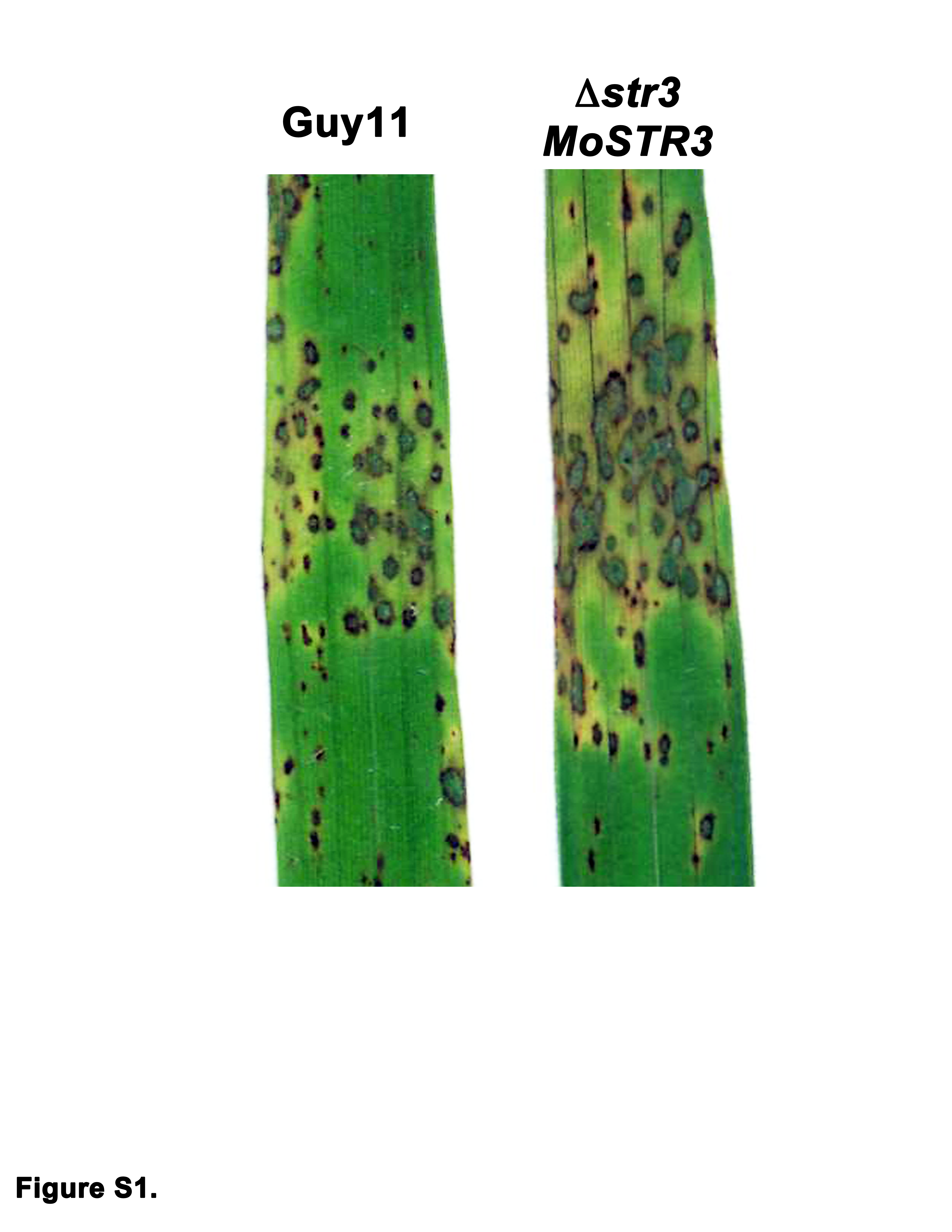

Supplement: Figure S1 — Δ str3 strains complemented with MoSTR3 are restored for pathogenicity. Spores of wild type Guy11 strains and Δstr3 MoSTR3 complementation strains were applied to three-week old rice plants of the susceptible cultivar CO-39 at a rate of 5×10^4^ spores ml−1. Images were taken after 144 hpi. (TIF) [file pone.0047392.s001.tif]
